# Cell Transport Prompts the Performance of Low-Voltage Electroporation for Cell Inactivation

**DOI:** 10.1038/s41598-018-34027-0

**Published:** 2018-10-25

**Authors:** Zheng-Yang Huo, Guo-Qiang Li, Tong Yu, Chao Feng, Yun Lu, Yin-Hu Wu, Cecilia Yu, Xing Xie, Hong-Ying Hu

**Affiliations:** 1 Environmental Simulation and Pollution Control State Key Joint Laboratory, State Environmental Protection Key Laboratory of Microorganism Application and Risk Control (SMARC), School of Environment, Tsinghua University, Beijing, 100084 PR China; 2 Institute for Advanced Study, Tsinghua University, Beijing, 100084 PR China; 3 School of Civil and Environmental Engineering, Georgia Institute of Technology, Atlanta, Georgia 30332 United States; 4 Shenzhen Environmental Science and New Energy Technology Engineering Laboratory, Tsinghua-Berkeley Shenzhen Institute, Shenzhen, 518055 PR China

## Abstract

The inactivation of pathogens in liquids has broad applications, ranging from water disinfection to food pasteurization. However, common cell inactivation methods (*e.g*., chlorination, ultraviolet radiation and thermal treatment) have significant drawbacks such as carcinogenic byproduct formation, energy intensiveness and/or nutrient structure destruction. Here, we fabricated a new approach to address these challenges by applying a low-voltage electroporation disinfection cell (EDC) and investigate the critical mechanisms of cell transport to allow high inactivation performance. The EDC prototypes were equipped with two one-dimensional (1D) nanostructure-assisted electrodes that enabled high electric field strength (>107 V m^−1^) near the electrode surface with a low applied voltage (1 V). We have identified that during electroporation disinfection, electrophoresis, dielectrophoresis and hydraulic flow are the three major mechanisms which transport cells into the vicinity of the electrode surface to achieve superior disinfection performance. The EDC treated 70 ml of bacteria sample with an initial cell concentration of 10^7^ CFU ml^−1^ and achieved complete bacteria inactivation (survival rate <0.00001%; no live bacteria detected). Our findings will help to establish a foundation for the future development and implementation of low-voltage electroporation for cell inactivation.

## Introduction

Inactivating pathogenic cells in liquids is a general process that has been broadly applied in the food and water industry^1–3^: *e.g*., 164.8 million tons of milk is pasteurized in the European Union per year, and more than 20,000 plants in the USA are performing drinking-water (29.4 billion gallons) and wastewater (32.1 billion gallons) disinfection every day^4,5^. Common cell inactivation methods include chlorination, ozonization, ultraviolet radiation, and thermal treatment. All of these processes can achieve high cell inactivation efficiency in certain conditions, but they suffer from inevitable formation of carcinogenic disinfection byproducts, intensive consumption of energy, regrowth of pathogens and/or destruction of nutrient structures^6,7^. In order to find a better cell inactivation method, scientists have explored many different approaches^8–10^, one of which is based on electroporation. Electroporation has been widely used in several areas of medicine for delivering DNA and/or proteins into cells^11,12^. When cells are exposed to an electric field with sufficiently high strength, a rapid and large increase in cell membrane permeability may occur. If the electric field strength is high enough (>10^7^ V m^−1^), irreversible electroporation occurs on the cell membrane, resulting in cell inactivation. Because electroporation is a physical phenomenon, cell inactivation by electroporation gains the advantages of high throughput, no chemical usage and outstanding efficacy to all pathogens^11,13–15^. However, high energy consumption (>10 kJ per liter of the liquid sample) and the technical challenges associated with generating a strong electric field strength without arcing have been major obstacles to the large-scale implementation of electroporation for cell inactivation^11,13^.

To overcome these limitations, conductive one-dimensional (1D) nanostructures have been introduced to enable cell inactivation by electroporation with low applied voltages (<20 V)^16–18^. When 1D nanostructures are inserted into a region of a uniform electric field, the electric field strength near the tips (*E*_*tip*_) of the 1D nanostructures can be enhanced with a degree roughly estimated by equations (1) and (2):

1 $$\frac{{E}_{{\rm{tip}}}}{{E}_{0}}={\rm{\alpha }}\cdot \frac{{l}_{{\rm{rod}}}}{{d}_{{\rm{rod}}}}$$

2 $${E}_{0}=\frac{U}{d}$$

where *E*_0_ is the strength of the original uniform electric field, *α* is a constant, *l*_*rod*_ is the length of the rod, *d*_*rod*_ is the diameter of the rod, *U* is the applied voltage and *d* is the distance between two electrodes^19^. Because 1D nanostructures have high aspect ratios (*l*_rod_*/d*_rod_), *E*_*tip*_ can be several orders of magnitude stronger than *E*_0_^19,20^. Therefore, the 1D nanostructures provide exceedingly high electric field strength near the tips to achieve electroporation for cell inactivation with low applied voltages^19,20^. Taking advantage of this effect, low-voltage electroporation devices have been introduced to achieve high disinfection performance with low energy consumption (<100 mJ per liter of water sample treated)^16,17,21^.

According to the theoretical analysis and the computational simulation, areas with sufficiently high electric field strength (>10^7^ V m^−1^) for irreversible cell electroporation are limited to very small regions within a few μm from the tips of the nanostructures^9^. It is therefore critical to transport target cells towards the tips to enable effective inactivation. Hydraulic flow contributes to cell transport. However, according to the previous study, considering the fast flow rate (contact time <10 s) and relatively large pore size of the electrode (~500 μm)^22^, it is not likely that the majority of the cells can be transported into these regions merely by the hydraulic flow. Thus, other mechanisms must provide cell transport to the tips. In the presence of an electric field, two processes that could cause cell transport are electrophoresis and dielectrophoresis^23,24^. Electrophoresis is the motion of charged particles caused by electrostatic force when exposed to an electric field^25,26^. Dielectrophoresis will transport a dielectric particle (*e.g*., cell) when it is polarized in a non-uniform electric field. However, how these mechanisms contribute to cell transport during low-voltage electroporation is still not fully understood.

Here in this study, we have constructed a 1D nanostructure-assisted low-voltage electroporation disinfection cell (EDC) which allows efficient, chemical free and high-throughput cell inactivation. During electroporation disinfection, we found that cells were transported into the vicinity of the electrode surface to achieve superior disinfection performance (survival rate <0.00001%; no live bacteria detected). The three critical transport mechanisms were electrophoresis, dielectrophoresis, and hydraulic flow. These findings bring the EDC one step closer to real-world applications which can substantially change the current production methods in the food and water industry.

## Results and Discussion

### Electroporation disinfection cell (EDC) fabrication and its disinfection performance

**Figure 1:**
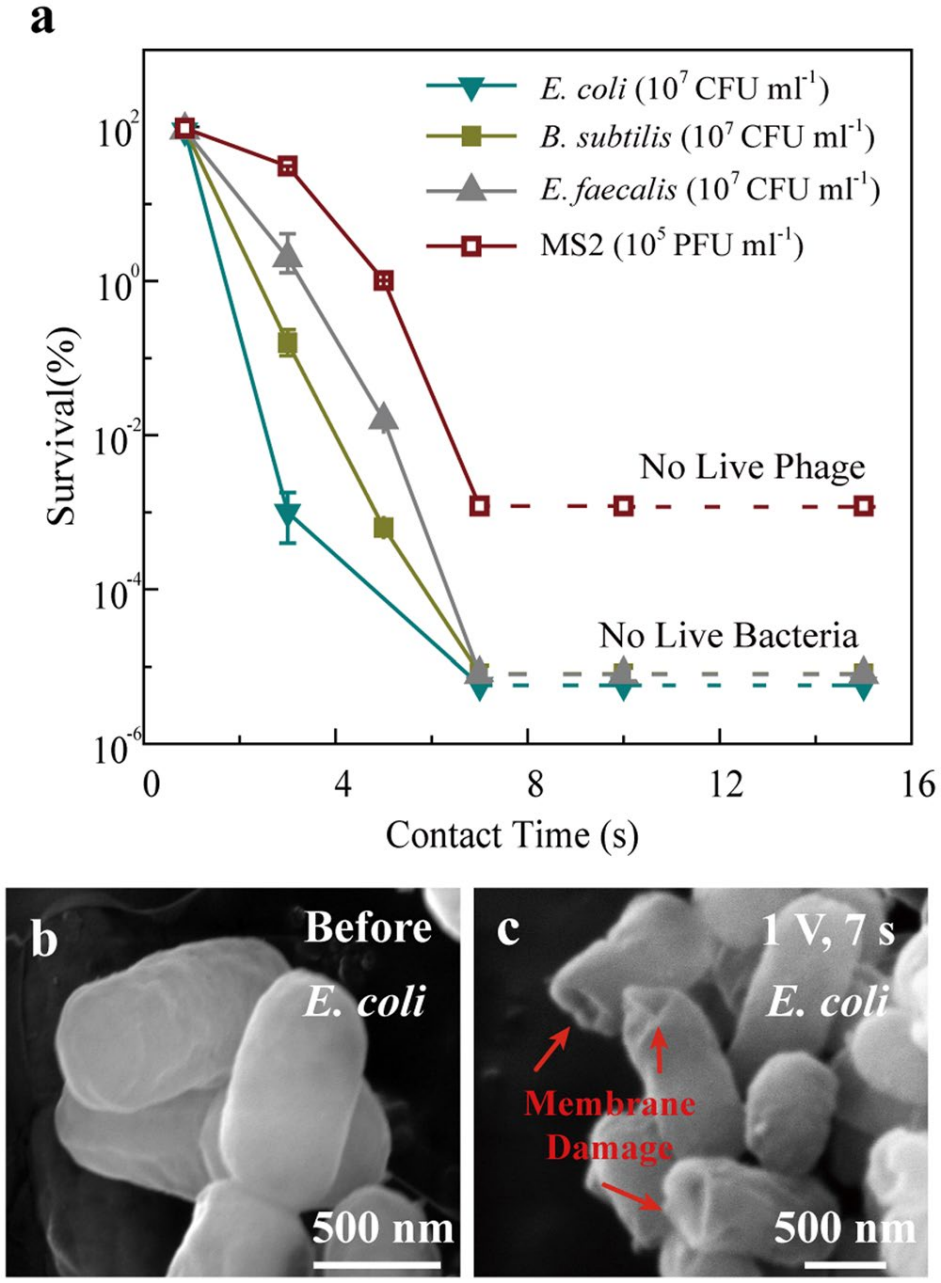
Cell inactivation by the EDC at 1 V applied voltage and mechanism analysis. (**a**) Survival rate of *E. coli*, *B. subtilis*, *E. faecalis*, and MS2 after EDC operation with a fixed applied voltage (1 V) and different contact times (from 1 to 15). Dashed lines indicate that all bacteria or phages were inactivated and no live bacteria or phages were detected. (**b**,**c**) SEM images of *E. coli* before (**b**) and after (**c**) 1 V, 7 s EDC operation.

To demonstrate the outstanding performance of 1D nanostructure-assisted low-voltage electroporation for cell inactivation, we developed an electroporation disinfection cell (EDC) based on our previous study (Fig. S1)^22,27^. The electrodes applied in the EDC were copper foams assisted with copper oxide nanowires (CuONW-Cu), which were synthesized through a simple one-step thermal oxidation approach^22^. An EDC was built with two such CuONW-Cu electrodes. The water sample containing bacteria flowed the electrode with the desired flow rate and the voltage (1 V) was applied at the same time (Fig. S1). Bacteria samples (70 ml) with an initial cell concentration of 10^7^ CFU ml^−1^ were treated by EDC. As shown in Fig. 1a, EDC achieved superior disinfection performance (no live bacteria detected) for all model microorganisms (*Escherichia coli, Bacillus subtilis, Enterococcus faecalis, and* MS2) when the contact time was longer than 7 s with an applied voltage of 1 V. At such conditions, the EDC can at least enabled complete disinfection (survival rate <0.00001%; no live bacteria detected) for 10 min (corresponding to ~70 ml of water with bacteria concentration of 10^7^ CFU ml^−1^; Fig. S2). The solution conductivity was fixed at 200 μS cm^−1^ to represent natural waters. Without the applied voltage, no bacteria inactivation occurred when the bacteria sample passed through the EDC with contact time from 1 to 15 s, indicating that the applied voltage is necessary for EDC operation (Fig. S3). Scanning electron microscope (SEM) images indicated the mechanism of electroporation disinfection. Compared to untreated bacteria, whose cell membranes were complete and smooth (Fig. 1b), the treated bacteria had obvious electroporation holes on the surface (Fig. 1c), indicating lethal membrane damage. The concentration of Cu in the effluent was less than 500 ug l^−1^, which was nonlethal for bacteria (Fig. S4). This indicated that the dissolved Cu was not able to kill the bacteria during EDC operation.

### Demonstration of critical cell transporting mechanisms in nanowire-assisted electroporation

**Figure 2:**
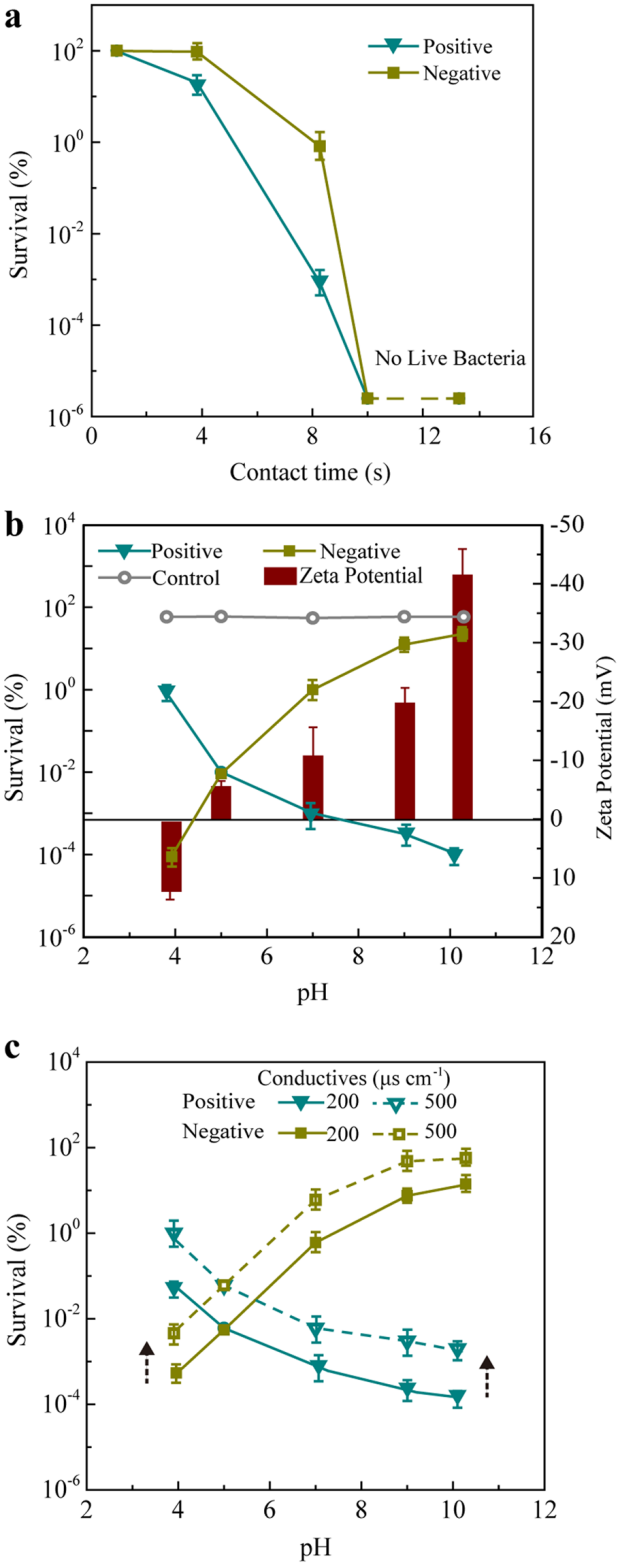
Demonstration of electrophoresis and dielectrophoresis in nanowire-assisted electroporation. (**a**) Survival rate of *E. coli* treated by the positive or negative electrode of an EDC with different contact times at pH = 7. Dashed lines indicated that all bacteria were inactivated and no live bacteria were detected. (**b**) Survival rate of *E. coli* treated by the positive or negative electrode of an EDC varying with pH (from 4 to 10) and the zeta potential of *E. coli* at corresponding pH values. (**c**) Survival rate of *E. coli* treated by the positive or negative electrode in media with *σ*_m_ of 200 and 500 μS cm^−1^ at different pH.

To demonstrate how electrophoresis, dielectrophoresis, and hydraulic flow transport cells to the electrode surface during EDC operation to achieve high-performance cell inactivation, we built EDCs with only one CuONW-Cu electrode, either as a positive or negative electrode. The other electrode was a copper-oxide-nanoparticle assisted copper foam (CuONP-Cu) (Fig. S5), which had been demonstrated to be ineffective for cell inactivation^17^. The performance of these EDC-treated *E. coli* samples with neutral pH is shown in Fig. 2a. The disinfection performance was significantly better when CuONW-Cu was applied as the positive electrode. We speculated that this obvious difference was caused by electrophoresis, which would send the *E. coli* towards the positive electrode since *E. coli* have negative surface charges when suspended in water with neutral pH^28,29^.

To verify this hypothesis, we further tested the inactivation efficiency of the EDC (1 V, 8 s) using water samples with different pH values. When the surrounding pH increased from 4 to 10, the surface charge of *E. coli* changed gradually from positive to negative, indicated by the change in zeta potential from 13 mV to −40 mV (Fig. 2b). As shown in Fig. 2b, the inactivation efficiency of EDCs increases with pH (survival rate decreasing from 1% to 0.0001%) when CuONW-Cu is the positive electrode but decreases with pH (survival rate increasing from 0.0001% to 95%) when CuONW-Cu is the negative electrode. Cell inactivation was therefore facilitated when the effective electrode and the *E. coli* carried opposite charges, and the electrophoretic force pushed the *E. coli* towards the effective electrode. When the surface charge of *E. coli* was approximately neutral, theoretically at pH 4.6^29^, the electrophoretic force was negligible and the inactivation efficiency of EDCs was therefore similar regardless of whether CuONW-Cu was used as the positive or negative electrode (Figs 2b and S6). More direct evidence of electrophoresis-driven cell transport could be collected by studying the cell attachment on the electrodes. The results of DNA mass measurement, SEM, and a staining test all indicated that significantly more *E. coli* were attached to the positive or negative electrode when they had opposite surface charges (Figs S7 and S8). All of these results supported that during the EDC operation, electrophoresis was an important mechanism for cell transport. This can also be confirmed by the impact of pH during EDC operation in our early study: the disinfection efficiency of the EDC was lowered at pH ~5^27^. In addition, during the 10-min EDC operation, at pH of 7, the total DNA mass in the effluent was measured as 55 mg, which was much higher than the attached DNA mass (2.5 mg; Fig. S7). Attribute to the inactivation mechanism of electroporation, cells were not necessary to contact with the electrodes during the electroporation process, thus most of the dead cells passed through the device. Only small amount of the cells were attached to the electrode. The previous study also showed that the optical density at 670 nm wavelength of bacteria samples before and after EDC operation remained similar^16^. This also indicated that the majority of bacteria passed through the device. With the relatively low conductivity, those attached cells would not significantly impact the electric field enhancement effect introduced by the nanowires. This was because the degree of enhancement of the electric field strength near the tip is determined by the aspect ratio of the conductive nanostructure^20^.

Although electrophoresis was confirmed to be the major mechanism for cell transport during EDC operation when the electrophoretic force was negligible at pH 4.6, the *E. coli* inactivation efficiency (0.01% survival rate) was still significant at 1 V, 8 s operation (Fig. S6). Therefore, there must exist driving forces other than electrophoresis that can transport *E. coli* to the CuONW-Cu electrode surface to achieve cell inactivation. Besides the possible physical transport mechanism caused by hydraulic flow, dielectrophoresis, another driving force, may also transport the cells. Dielectrophoretic forces are able to transport dielectric particles (*e.g*., cells) in a non-uniform electric field.

When exposed to an electric field, the charges of a dielectric particle are rearranged. Positive charges are induced on one side with the same amount of negative charges on the opposite side. If the electric field is non-uniform, the force on one side is greater than that on the other side, resulting in a net dielectrophoretic force (*F*_DEP_) that can move the particle. For spherical particles, according to the theory of dielectrophoresis, *F*_*DEP*_ is determined by equation (3):

3 $${F}_{{\rm{DEP}}}=2\cdot {\rm{\pi }}\cdot {\varepsilon }_{0}\cdot {\varepsilon }_{{\rm{m}}}\cdot {r}^{3}\cdot {f}_{{\rm{CM}}}\cdot \nabla {E}^{2}$$

where *E* is the strength of the non-uniform electric field, *r* is the radius of the particle, *ε*_0_ is the absolute dielectric constant, *ε*_*m*_ is the relative dielectric constant of the suspending fluid and *f*_CM_ is the Clausius-Mossotti factor, which can be further determined by equation (4):

4 $${f}_{{\rm{CM}}}=\frac{{\sigma }_{{\rm{c}}}-{\sigma }_{{\rm{m}}}}{{\sigma }_{{\rm{c}}}+2{\sigma }_{{\rm{m}}}}$$

where *σ*_c_ and *σ*_m_ are the electrical conductivities of the particle and fluid, respectively^30^. Depending on the relative conductivities of the particle and surrounding fluid, *f*_CM_ has a value between −0.5 and 1. If *f*_CM_ >0 (*i.e*., *σ*_c_ > *σ*_m_), the particle experiences positive *F*_*DEP*_ and is transported to the high *E* region (towards the electrode surface); if *f*_CM_ < 0 (*i.e*., *σ*_c_ < *σ*_m_), the particle experiences negative *F*_*DEP*_ and is transported to the low *E* region (away from the electrode surface)^28,31–33^.

To investigate and calculate the *F*_*DEP*_ for the *E. coli* in water easily, we simplified the *E. coli* cells as spherical particles. Considering *E. coli* as a dielectric particle, its *σ*_c_ is ~410 μS cm^−1^ ^28^. To test the hypothesis that in addition to hydraulic flow and electrophoresis, dielectrophoresis also contributes to cell transport during EDC operation, we performed experiments using media with a *σ*_m_ of 500 μS cm^−1^ at different pH values with 1 V applied voltage and 8 s of contact time. The cell inactivation results supported our hypothesis. As shown in Fig. 2c, with 1 V, 8 s operation, all bacteria survival rates were around 1 order of magnitude higher than the previous bacteria survival rates with lower *σ*_m_ (~200 μS cm^−1^) for both positive and negative electrodes. Under such conditions, *f*_CM_ < 0 and theoretically, the negative *F*_DEP_ repelled *E. coli* away from the electrode surface, which resulted in less cell inactivation. The rest of the inactivated cells were believed to be killed as a result of hydraulic flow transport.

Considering the pH of typical water samples (~7) and the conductivity (~200 μS cm^−1^), low-voltage electroporation with high efficiency can be realized under these conditions. Cells could be inactivated with the help of three different cell transport mechanisms: electrophoresis, dielectrophoresis and hydraulic flow. Each mechanism had its respective contribution to cell inactivation. When changing the surface charge of *E. coli* to neutral by adjusting pH, the impact of electrophoresis would be excluded and the cell survival rate increased by 3 orders of magnitude to 99.99% (Fig. S9). At this pH, if the medium conductivity was increased above the cell conductivity, the dielectrophoresis could repel the cell away from the electrode and the *E. coli* survival rate further increased another 2 orders of magnitude to 99% (Fig. S9). However, the impact of hydraulic flow was significant but difficult to quantify because of the complicated flow regime which existed inside electrodes with a foam structure.

### Confirmation of electrophoresis and dielectrophoresis during EDC operation in the batch mode

**Figure 3:**
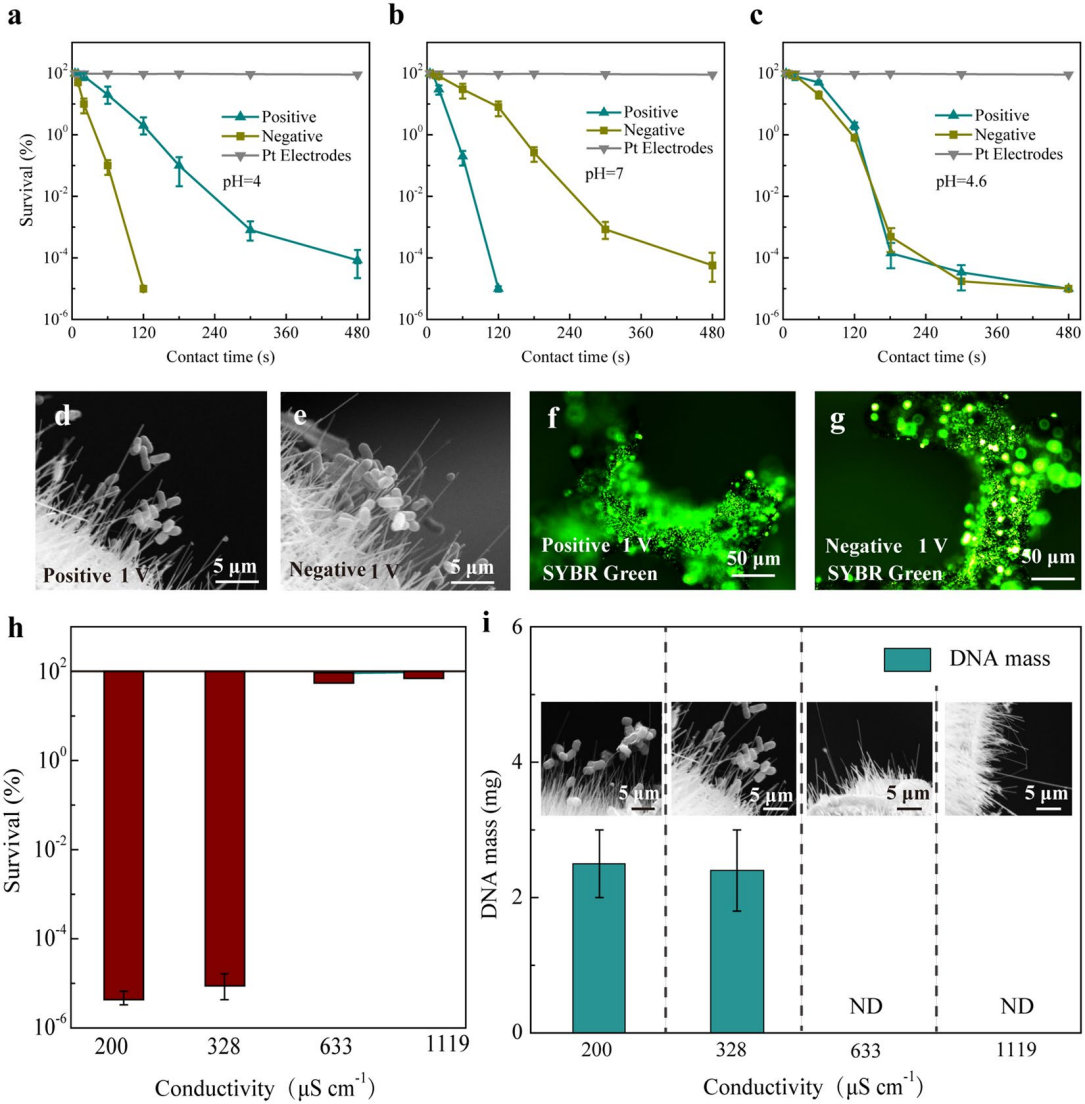
Demonstration of electrophoresis and dielectrophoresis during EDC operation in the batch mode. (**a**–**c**) Survival rate of *E. coli* treated by CuONW-Cu electrodes and Pt plate electrodes varying with contact time. The distance between the electrodes was 2 mm and the pH was fixed at (**a**) 4, (**b**) 7 and (**c**) 4.6. (**d**,**e**) SEM images of the positive (**d**) and negative (**e**) electrodes after EDC operation at pH of 4.6. (**f**,**g**) Fluorescence microscope images of the positive (**f**) and negative electrodes (**g**) after EDC operation at pH of 4.6. (**h**) Survival rate of *E. coli* treated by CuONW-Cu electrodes varying with conductivity at pH of 4.6. (**i**) Mass of DNA extracted from attached cells on the CuONW-Cu electrodes and SEM images of CuONW-Cu electrodes after EDC operation for 10 min with different conductivity at pH of 4.6.

Considering that the impact of hydraulic flow is difficult to quantify and may be a confounding factor in electrophoresis or dielectrophoresis transport, we employed a new EDC with only one effective CuONW-Cu electrode and performed low-voltage electroporation in the batch mode (Fig. S10). In this way, cell transport by hydraulic flow could be minimized and the impact of electric transport forces without hydraulic flow can be investigated. The distance between the electrodes and the applied voltage were set to 2 mm and 1 V, respectively. The pH values of the water samples were fixed at 4, 4.6 and 7. When using flat Pt plate electrodes, a uniform electric field was generated between the electrodes. The electric field strength was approximately 100 V m^−1^, which was much lower than that required for irreversible electroporation (>10^7^ V m^−1^). Consequently, no significant *E. coli* inactivation was observed for all samples at different pH (Fig. 3a–c). However, when CuONW-Cu electrodes were applied, *E. coli* were gradually inactivated and the survival rate dropped to 0.00001% after an 8-min operation (Fig. 3a–c). These results confirmed our speculation that there were driving forces other than hydraulic flow transporting *E. coli* to the CuONW-Cu electrode surface to realize cell inactivation. The disinfection performance of either the positive or negative electrode at different pH (which determined the cell surface charges) indicated that when the effective electrode and *E. coli* carried opposite charges, cell inactivation could be promoted (Fig. 3a–c). These results also supported the hypothesis that electrophoresis sent cells to the effective electrode and facilitated the electroporation disinfection performance.

While electrophoresis was negligible at pH 4.6, it was noteworthy that the cells were still inactivated completely for either positive or negative electrode after 5 min operation (Fig. 3c). SEM images (Figs 3d,e and S11) and a staining test (Figs 3f,g and S12) showed obvious attachment of *E. coli* on both the positive and negative electrodes after operation. Thus we believed that besides electrophoresis, dielectrophoresis also contributed to cell transport during batch mode EDC treatment. We further performed experiments using media with different *σ*_m_ values (200, 328, 633, and 1119 μS cm^−1^). As shown in Fig. 3h, *E. coli* are effectively inactivated after 5 min treatment only when *σ*_c_ (410 μS cm^−1^) > *σ*_m_ (200 and 328 μS cm^−1^). Under such conditions, *f*_CM_ > 0 and *E. coli* were transported by positive *F*_DEP_ to the electrode surface with high *E*, resulting in cell inactivation. This explanation was also supported by the results of DNA mass measurement, SEM and a staining test (Figs 3i and S13), which showed cell attachment on the electrodes when *σ*_c_ > *σ*_m_. In contrast, when *σ*_c_ (410 μS cm^−1^) < *σ*_m_ (633 and 1119 μS cm^−1^), no obvious cell attachment and inactivation were observed (Figs 3h,i and S13). These results confirmed that dielectrophoresis also played an important role in cell transport during EDC operation.

### Simulation of major forces affecting cell transport during EDC operation

To quantify the major forces, computational analysis was performed. A simplified EDC configuration was applied for the simulation. Two nanowire-assisted plate electrodes were set at 100 μm apart, and the voltage was set at 1 V. The diameter and height of the nanowires were 10 nm and 15 μm, respectively. For each millimeter, there were 100 nanowires grown on the electrodes. Water contaminated by *E. coli* (pH of 7 and conductivity of 200 μS cm^−1^ for typical freshwater samples) passed through the space between the two parallel electrodes as laminar flow (Fig. S14a). The electric field distribution between the electrodes was simulated (Fig. S14b), and the electric field strength at different distances from the nanowire tip structure was calculated according to the simulation results (Fig. S14c). Under these conditions, the effects of the hydraulic force on *E. coli* could be ignored, and the other forces (electrophoresis, dielectrophoresis, gravity, buoyancy and Brownian force) could all be estimated (see details in additional discussion 1).

**Figure 4:**
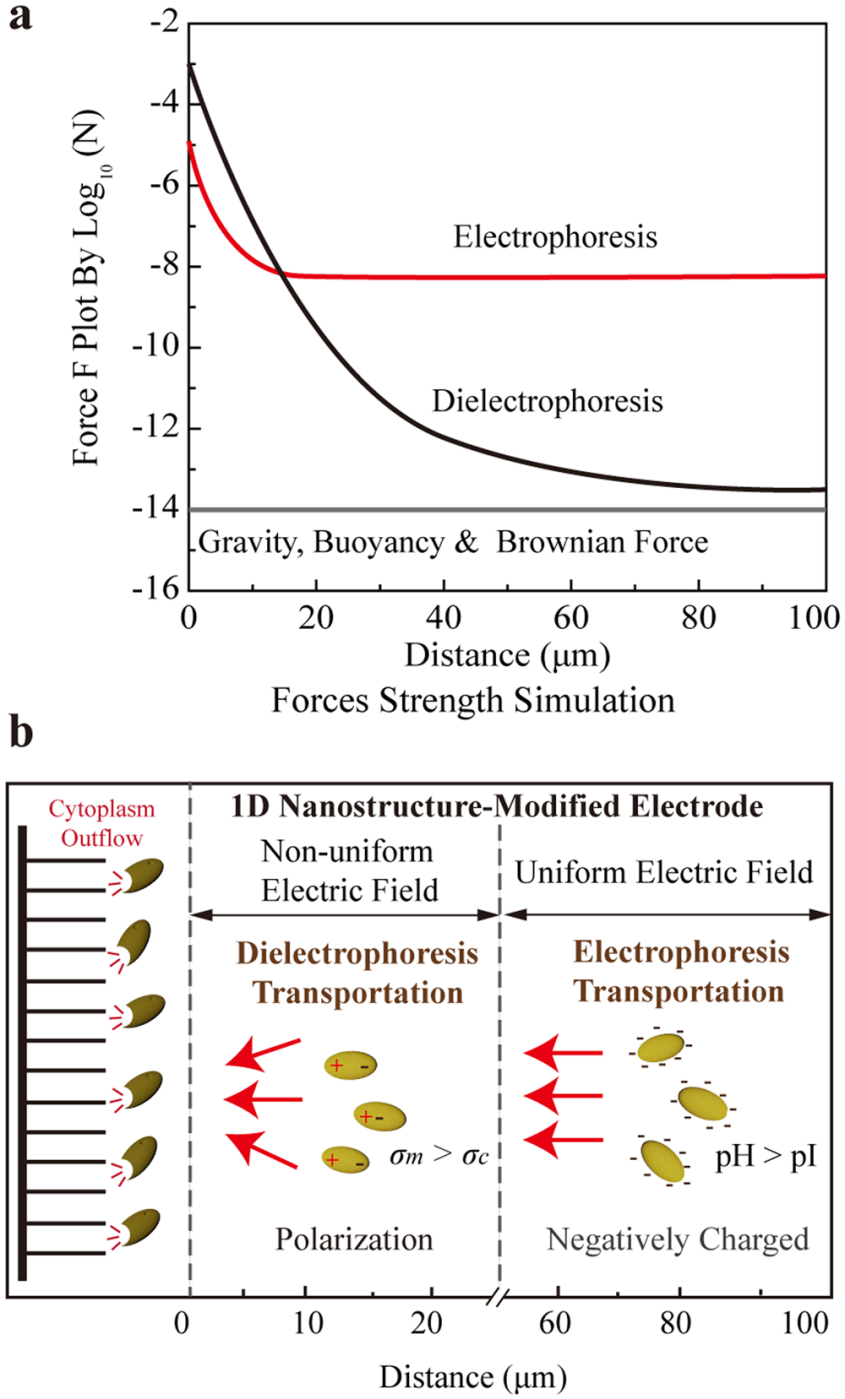
Simulation results during EDC operation. (**a**) Simulation of major forces affecting cell transport during EDC operation. (**b**) Illustration summarizing the mechanisms of electrophoresis and dielectrophoresis transporting the cell towards electrodes.

According to the electric field strength distribution, electrophoresis (*F*_*EP*_) and dielectrophoresis (*F*_*DEP*_) were determined by equations (5) and (3), respectively:

5 $${F}_{EP}=E\times {q}_{cell}$$

where *E* is the strength of the electric field and ${q}_{cell}$ is the charge carried by the cell (additional discussion 1). As shown in Fig. 4a, the simulation results indicated that both electrophoretic and dielectrophoretic forces were several orders of magnitude higher than the other forces, thus playing a more significant role in cell transport. Electrophoretic forces predominated when *E. coli* were relatively far away from the electrode surface (>20 μm) (Fig. 4a). In this region, electric field strength was mainly determined by the applied voltage and electrode distance with low spatial gradient, resulting in a much lower dielectrophoretic force. The electric field strength was enhanced abruptly near the electrode surface (<20 μm) because of the nanowires. Accordingly, both electrophoretic and dielectrophoretic forces increased significantly, with the dielectrophoretic force gradually surpassing the electrophoretic force to become the dominant factor (Fig. 4a). Therefore, a typical cell transport process was as follows (Fig. 4b): when *E. coli* passed through the electrodes, the negatively charged cells were first transported by the electrophoretic force into the uniform electric field. As the cells approached the surface of the electrodes (within 20 μm) where the electric field was enhanced by the nanowires, the dielectrophoretic force increased remarkably and became the major driving force. Transported by those two forces, the cells were then attracted close to the electrode surface (~μm) with the enhanced electric field, and inactivation occurred subsequently. Based on the results of electric field distribution simulation (Fig. S14b), the region with strong electric field strength (>10^6^ V/m; enabling electroporation cell inactivation) has a large diameter (>10 μm). Considering the relativity small diameter of bacteria (<0.5 μm), the region with strong electric field strength will not be blocked by the cells. This result also agreed with the discussion that most of the dead cells passed through the EDC without obvious sticking.

### Discussion and outlook

The EDC achieved superior disinfection efficiency (survival rate <0.00001%; no live bacteria detected) with a low applied voltage (1 V) and short contact time (7 s). Although the hydraulic flow (advection) can send the bacteria to the electrode surface where high electric field strength exists, electrophoresis and dielectrophoresis contributed more to transport the cells. When the impact of electrophoresis was excluded, the cell survival rate increased by 3 orders of magnitude to 99.99%, and when the impact of dielectrophoresis was excluded, the *E. coli* survival rate further increased another 2 orders of magnitude (Fig. S9). This indicated that the contribution of electrophoresis to cell transport was higher than that of dielectrophoresis during EDC operation. As the applied voltage increased, the contribution of electrophoresis also increased because of the higher electric field strength (equation (5)), whereas the dielectrophoretic forces did not change too much due to the unchanged spatial gradient (equation (3)). On the other hand, with high conductivity, the dielectrophoretic forces would not increase notably because that the *f*_CM_ could not change much (>−0.5) (equations (3) and (4)). It was, therefore, highly possible that when applied with high voltage and in the water with high conductivity, the increased electrophoretic forces could surpass the negative impact of dielectrophoretic forces and became the dominant contribution. This was also confirmed by our previous study. When applied with the increased voltage, the EDC still achieved complete disinfection (no live bacteria in the effluent) with high conductivity (8000 μS cm^−1^; corresponding to 100 mM of ionic strength)^27^.

Interestingly, when the impact of hydraulic flow was removed in the batch mode, the EDC still enabled complete cell inactivation (survival rate <0.00001% with no live bacteria detected; Fig. 4a,b). When eliminated the electrophoretic force (pH = 4.6) and decreased the dielectrophoretic force (*σ*_m_ = 633 μS cm^−1^), no obvious cell attachment on the electrode surface or inactivation was detected (Figs 4h,i and S13). This confirmed that electrophoresis and dielectrophoresis were the major cell transport mechanisms during the electroporation operation. Others transport mechanisms, such as electro-osmosis, might also contribute to the cell transport, whereas their effects were not significant^34^. On the other hand, during the electroporation disinfection process, the high electric field strength would directly inactivate the cell; therefore, even without reaching the electrode surface, cells were inactivated when they were sufficiently close to the nanowire (within several μm). Thus, the mechanism of cell transport to within 100 nm of the electrode surface *i.e*., cell transport through the electric double layer (usually less than 20 nm) or the diffusion boundary layer (usually less than 100 nm in our case) would not contribute significant during the disinfection process^35–37^.

The semi-quantitative analysis of the contribution of electrophoresis and dielectrophoresis during the nanowire-assisted electroporation disinfection could guide the designation of the real-world application. Taking into account the different attracting forces, the velocity of the cell transport in water could be calculated, and the cell transport time corresponding to different distances from the electrode could be simulated as well. Considering that the time for cell inactivation by electroporation was very short (<μs), the time for cell transport was thus the speed-limiting factor during the EDC operation. If the cell transport time was shorter than the contact time or the hydraulic retention time, all the bacteria will be transported to the region with enough electric field strength to achieve complete inactivation.

## Conclusion

In summary, the application of 1D nanostructure-assisted low-voltage electroporation allows efficient, reliable and high-throughput cell inactivation, which can substantially change the current production methods in the water industry. We have identified and characterized how cells were transported into the high-electric-field-strength zones enabling inactivation by three critical mechanisms: electrophoresis, dielectrophoresis and hydraulic flow. These findings can help to establish a theoretical foundation for the future development and implementation of EDCs.

## Methods

### Electrode fabrication, device construction, and electrode characterization

Copper foams (Zhuoer, China, pore size ~500 μm) were cut into cylinders with a diameter of 1 cm and a thickness of 0.5 cm. After being etched with 1 M hydrochloric acid (HCl, Sigma) to remove the oxide layer and washed three times with deionized (DI) water, the cylinders were heated in air at 400 °C for 120 min to grow copper oxide nanowires (CuONWs) or at 800 °C for 60 min for copper oxide nanoparticles (CuONPs). Two pieces of the modified copper foams were then fitted into a plexiglass coaxial electrode holder to prepare an EDC. The distance between the two copper foam electrodes was fixed at 2 mm.

The morphologies of CuONWs and CuONPs were characterized using a field emission scanning electron microscope (FEI-SEM, Zeiss, Ultra 60). CuONWs were also drop-cast onto a copper grid and then observed by a transmission electron microscope (TEM, JEOL, JEM-200CX).

### Water sample preparation for EDC operation

All solutions in this study were autoclaved (121 °C for 20 min) before being dosed with bacteria. Solutions were kept at a designated pH in a range from 4 to 10, which was adjusted using autoclaved (121 °C for 20 min) NaOH (0.1 M, Sigma) and HCl (0.1 M) as necessary. The conductivities of the solutions varied from 200 to 10^3^ μS cm^−1^ and were controlled using sodium chloride at room temperature.

Cell inactivation by EDC was demonstrated using three model bacteria and one model virus: *Escherichia coli* (ATCC 15597), *Enterococcus faecalis* (ATCC 19433), *Bacillus subtilis* (ATCC 6633) and MS2. Each bacteria sample was cultured to log phase (12 h) and then diluted to the designated solution (~10^7^ CFU ml^−1^). The MS2 was grown with the *E. coli* host on a shaker table set to 150 rpm at 37 °C for 24 h. MS2 was isolated and concentrated using the polyethylene glycol (PEG, Sigma) precipitation method. A solution of ∼10^5^ PFU ml^−1^ was prepared using normal saline solution (9.0 g l^−1^ sodium chloride, Sigma).

In the flowing mode, each water sample (70 ml) passed through the EDC device at different contact times with the designated pH and solution conductivity. Considering that the volume of the electrodes (φ1 cm × 1 cm) is 0.785 cm^3^, flow rates were kept in the range of 3.14–47.1 ml min^−1^, corresponding to contact times of 15 to 1 s. At the same time, a voltage of 1 V was applied to the device. In the batch mode, water samples (10 ml) containing bacteria (~10^7^ CFU ml^−1^) were added to a batch (15 ml) for different contact times (10 to 480 s), and the distance between electrodes was fixed at 2 mm. At the same time, a voltage of 1 V was applied to the device. Control experiments were conducted under the same conditions except using two Pt plate electrodes.

The bacterial concentrations of influents and effluents were measured using standard spread plating techniques and MS2 was enumerated using a double agar layer method^38,39^. Each sample was serially diluted and each dilution was plated in triplicate. All the results for each sample were averaged and the standard deviation was calculated. All plating was done within 3 h after the sample collection. Influents and effluents were compared to determine the inactivation efficiency.

### Measurement of zeta potential for bacteria

The prepared *E. coli* solution, which had been cultured to log phase (12 h), was diluted to a concentration of ~10^5^ CFU ml^−1^ in 10 mM NaCl solution with different pH values. After that, zeta potentials for bacteria under different pH were measured using Zetasizer Nano (BeckmanCoulter, DelsaNano C). The zeta potential measurement was repeated for 3 times. The results were averaged and the standard deviation was calculated.

### Bacteria sample preparation for SEM

Bacteria SEM images were taken by the SEM (Zeiss, Ultra 60). For SEM, the bacteria and electrode samples were pretreated with a fixing and critical point-drying process^40^. Specifically, bacteria samples after EDC operation were harvested by centrifuging at 14500 rpm for 15 minutes (HITACHI, RX2 series). Both the harvested bacteria samples and the treated electrodes were fixed in a solution containing 2% glutaraldehyde (Sigma) at 4 °C for 12 h. After washing with DI water for 5 min, the fixed samples were then dehydrated in increasing concentrations of ethanol solution (50, 70, 80, 90, and 100%, Sigma) and critical point dried in 100% ethanol with liquid CO_2_.

### Live/Dead staining experiment

Electrodes after EDC operation were washed with DI water for 5 min and soaked in 1 ml 0.1 M phosphate buffer solution (pH 7.3; Sigma). Live/Dead Baclight kit (Thermo Fisher) was used for the staining experiment. Equal volumes (2.5 μl) of SYTO 9 (0.6 mM) and PI (3 mM) dye solutions were added into the samples. Samples were stored in the dark for 30 min and washed with DI water for 3 times. After that, samples were examined using the fluorescent microscopy.

### Measurement of bacterial DNA mass

To measure the DNA mass of bacteria attached to the electrode, the electrodes after 10 min EDC operation (1 V, 8 s) were washed with DI water for 5 min and treated by ultrasound for 30 min to extract DNA from cells. To measure the DNA mass of bacteria, the water sample (~70 ml) after 10 min EDC operation (1 V, 8 s) was collected and treated by ultrasound for 30 min to extract DNA from cells. The extracted DNA was separated by centrifuging at 14500 rpm for 15 min (HITACHI, RX2 series) to leave the extracted DNA molecules in the supernatants. A volume of 100 μl of these supernatants was added to 2.5 ml 4, 6-diaminodino-w-phenylindole (DAPI) reagent (0.2 mg l^−1^ DAPI in 100 mM NaCl, 10 mM EDTA, 10 mM TRIS, pH 7, Sigma). Fluorescence of the samples was measured using a microplate reader (Molecular Devices, SpectraMax) (excitation wavelength fixed at 360 nm). The bacterial DNA concentration measurement was performed in triplicate and the results were analyzed.

### Cu measurement

The concentration of Cu in the effluents was measured by an inductively coupled plasma mass spectrometry (ICP-MS, Thermo Scientific, X Series 2). Treated water samples were filtered through 0.45 μm filters and stored in 1 M nitric acid before measurement. The Cu concentration was measured in triplicate. The results were averaged and the standard deviation was calculated.

### Electric field simulation

The electric field simulations were conducted using Matlab2014 (additional discussion 1). A simplified EDC configuration was applied for the simulation. Two nanowire-assisted plate electrodes were set as 100 μm apart and the voltage was 1 V. The density of CuONW distribution was 10^4^ nanowires per mm^2^. Water contaminated by *E. coli* (pH of 7 and conductivity of 200 μS cm^−1^ for typical freshwater samples) passed through the space between the two parallel electrodes as laminar flow (see detail parameters in Table S1).

## Electronic supplementary material

Supplementary Information
