# Supplementary material for: Cell Transport Prompts the Performance of Low-Voltage Electroporation for Cell Inactivation
Source: Sci Rep. 2018 Oct 25;8:15832. doi: 10.1038/s41598-018-34027-0 (PMC6202345; doi:10.1038/s41598-018-34027-0)
Supplement: Supplementary file 1 — Supplementary Information [file 41598_2018_34027_MOESM1_ESM.docx]

Supplementary information (SI):

Cell Transport Prompts the Performance of Low-Voltage Electroporation for Cell Inactivation †

Zheng-Yang Huo^ac^, Guo-Qiang Li^a^, Tong Yu^a^, Chao Feng^b^, Yun Lu^a^, Yin-Hu Wu^a^, Cecilia Yu^c^, Xing Xie^*c^, and Hong-Ying Hu^*ad^

^a^Environmental Simulation and Pollution Control State Key Joint Laboratory, State Environmental Protection Key Laboratory of Microorganism Application and Risk Control (SMARC), School of Environment, Tsinghua University, Beijing 100084, PR China. E-mail: [hyhu@tsinghua.edu.cn](mailto:hyhu@tsinghua.edu.cn); Tel: +86-10-6279-4005.

^b^Institute for Advanced Study, Tsinghua University, Beijing 100084, PR China.

^c^School of Civil and Environmental Engineering, Georgia Institute of Technology, Atlanta, Georgia 30332, United States. E-mail: xing.xie@ce.gatech.edu; Tel: +1-404-894-9723.

^d^Shenzhen Environmental Science and New Energy Technology Engineering Laboratory, Tsinghua-Berkeley Shenzhen Institute, Shenzhen 518055, PR China.

* Corresponding author:

Xing Xie: E-mail: xing.xie@ce.gatech.edu; Tel: +1-404-894-9723

Hong-Ying Hu: E-mail: [hyhu@tsinghua.edu.cn](mailto:hyhu@tsinghua.edu.cn); Tel: +86-10-6279-4005

**Supplementary Figures and Tables**

**
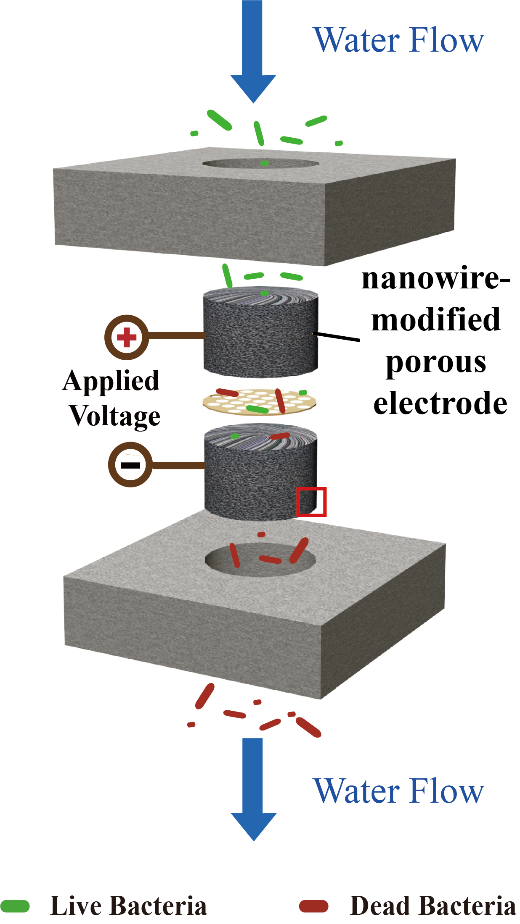
**

**Fig. S1. Schematic showing the construction of an EDC.**


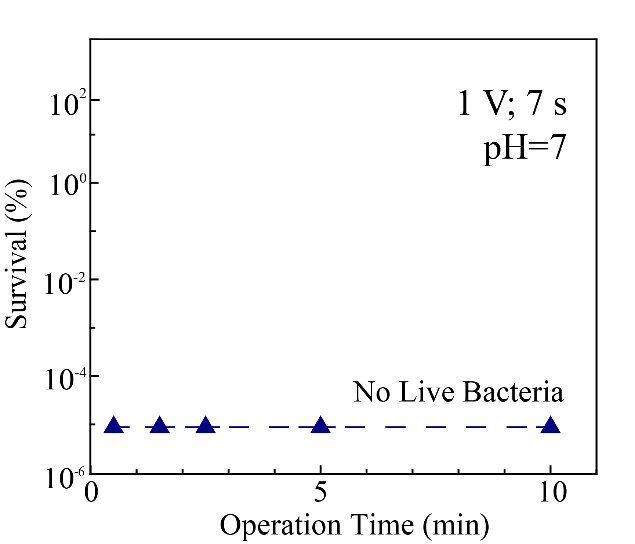


**Fig. S2. The disinfection efficiency of EDC during continuous operation.** The applied voltage was fixed at 1 V, the contact time was fixed at 7 s and pH was fixed at pH=7. Dashed lines indicate that all bacteria were inactivated and no live bacteria were detected.


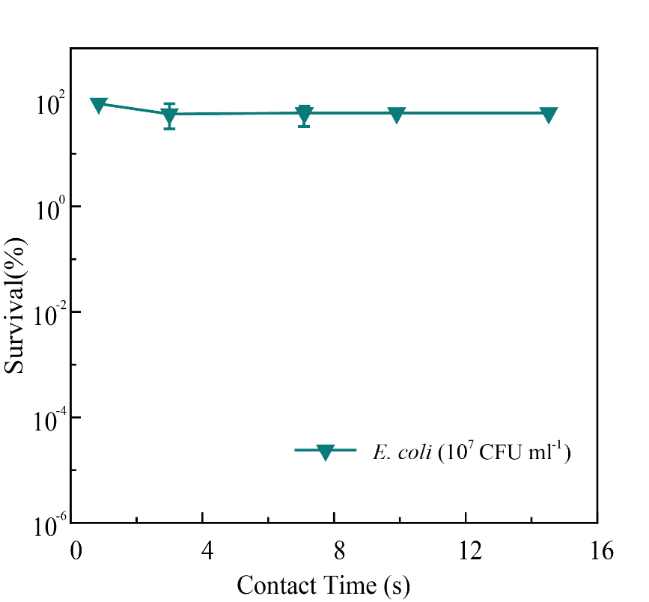


**Fig. S3. The disinfection performance of EDC with 0 V applied voltage and different contact times.**

**
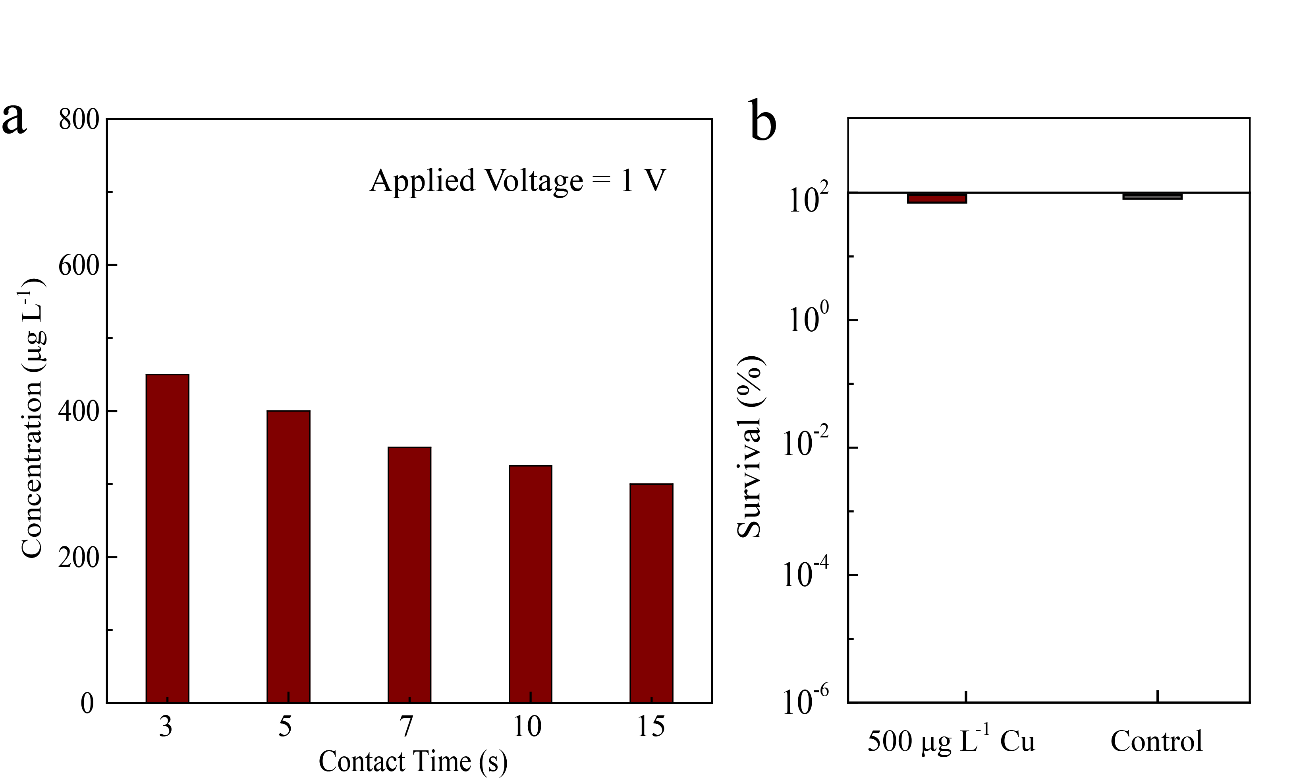
**

**Fig. S4. The concentration of Cu in the effluent after 1 V EDC operation with varying contact times and bacteria survival rate after being cultured in a designated concentration of Cu.** (**a**) The concentration of Cu in the effluent was less than 500 ug L^-1^ after 1 V EDC operation. (**b**) The bacteria survival rate after being cultured in a desired Cu concentration (500 μg L^-1^) at 37 ℃ for 24 h. Control sample is the bacteria survival rate after being cultured without Cu at 37 ℃ for 24 h. Almost all the bacteria survived after 24 h in 500 ug L^-1^ Cu^­^.


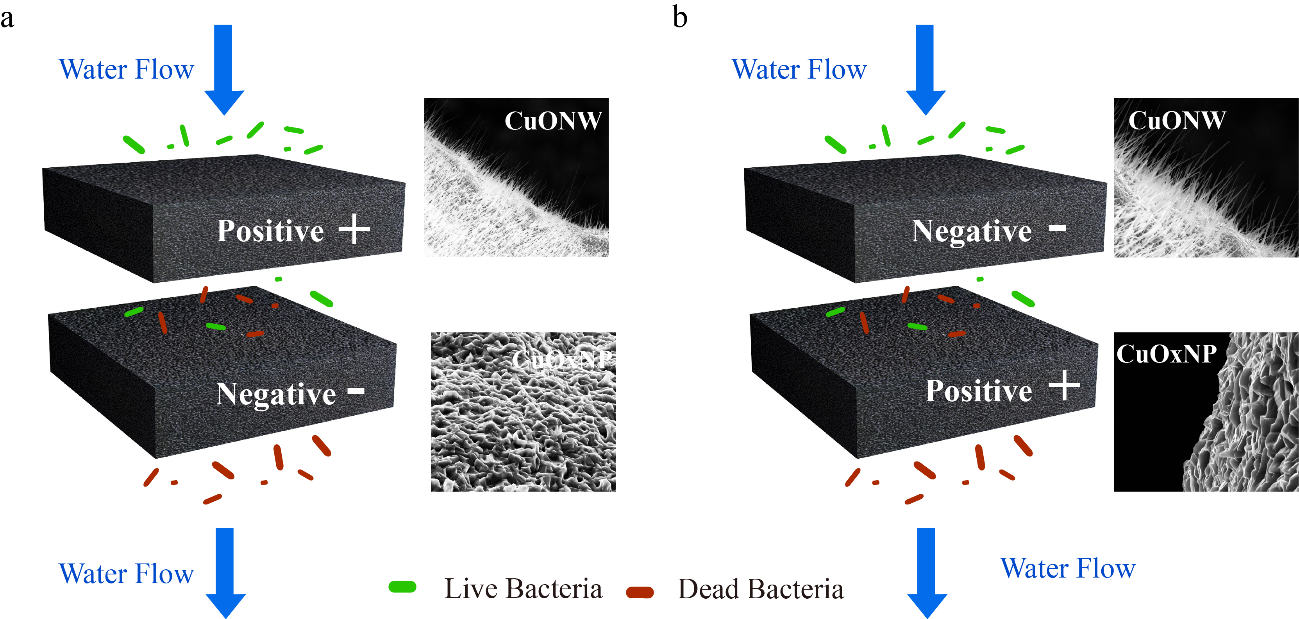


**Fig. S5.** **Demonstration of electrophoretic and dielectrophoretic forces for nanowire-assisted electroporation.** Schematics showing water samples passing vertically through the EDCs with only one CuONW-Cu electrode, either as a positive or negative electrode. The other electrode was a copper-oxide-nanoparticle assisted copper foam (CuONP-Cu) (**a**) The EDC with a positive electrode assisted with CuONW and negative electrode assisted with CuONP. (**b**) The EDC with a negative electrode assisted with CuONW and positive electrode assisted with CuONP.


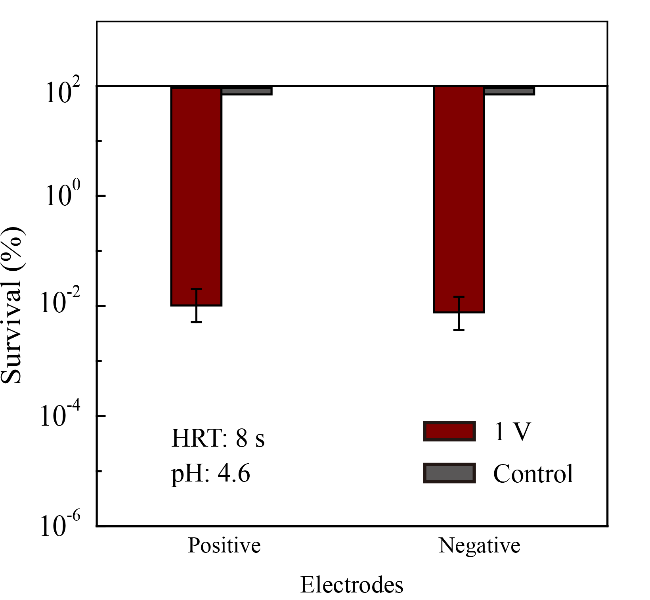


**Fig. S6.** **Demonstration of electrophoresis for nanowire-assisted electroporation.** Survival rate of *E. coli* treated by positive and negative electrodes respectively at 1 V, 8 s operation with pH of 4.6 (pI of *E. coli*).


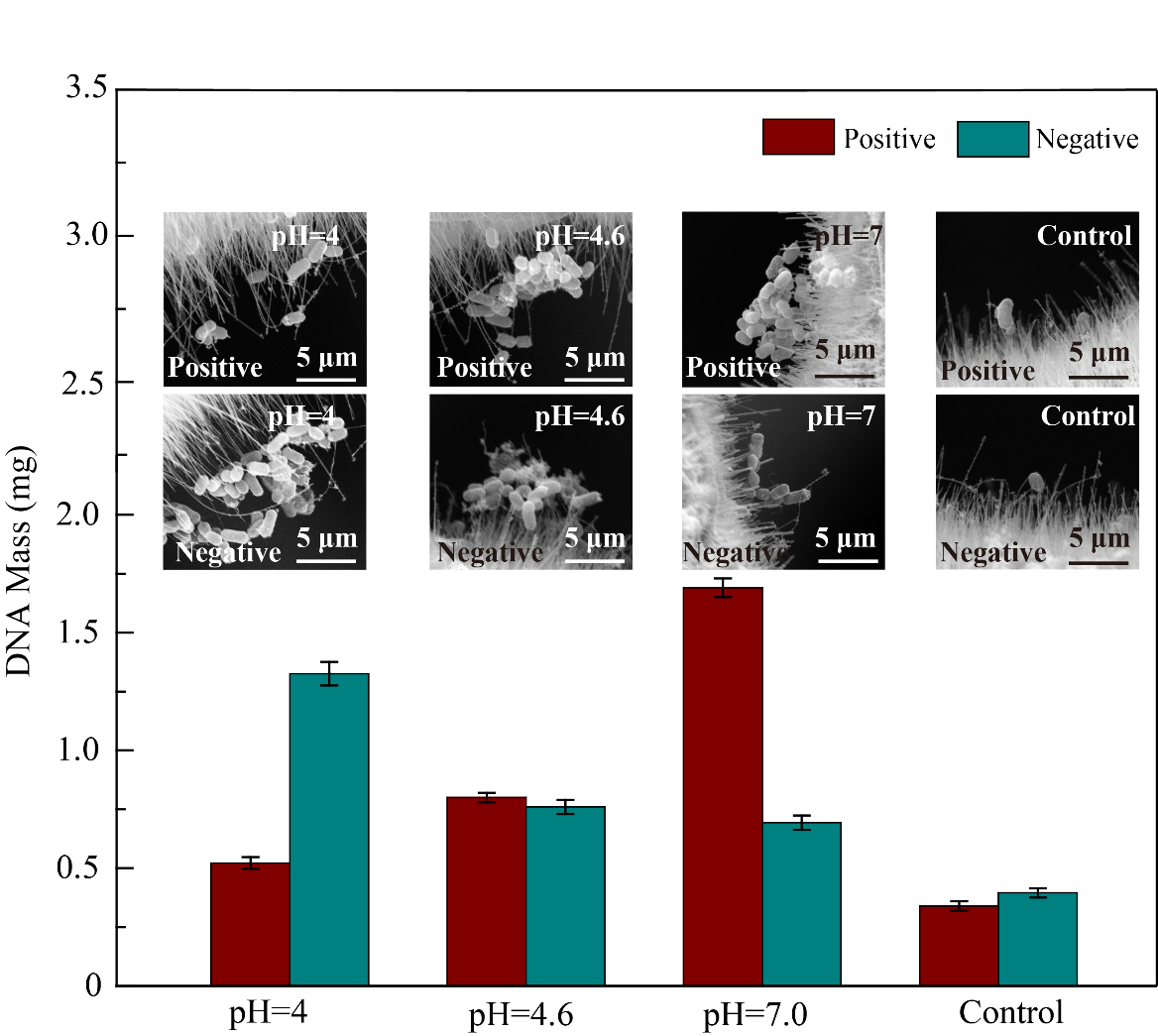


**Fig. S7. Electrophoretic force confirmation.** The mass of DNA extracted from the cells which were attracted to the electrodes. Control samples were treated by EDC without applied voltage. During the 20-min EDC operation, the total DNA mass in the effluent was measured as 55 mg at pH of 7.


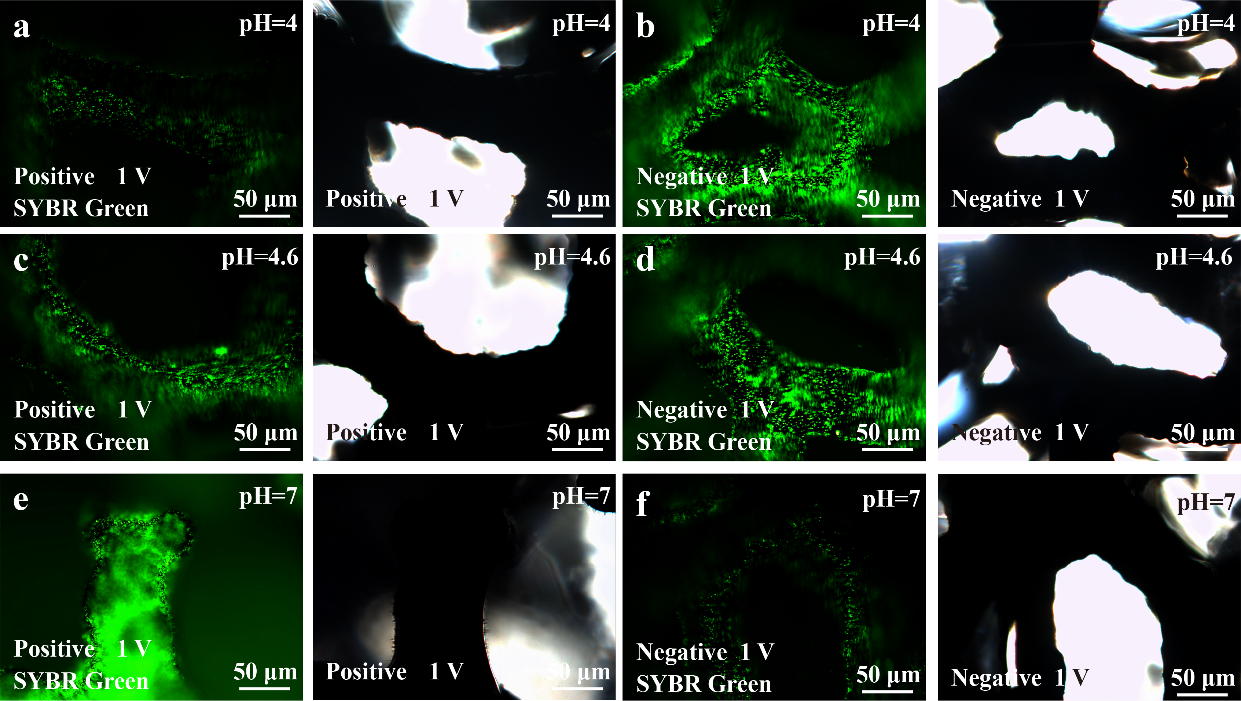


**Fig. S8. Electrophoretic force confirmation.** Fluorescence microscope and bright field images of the positive electrode (**a**) and negative electrode (**b**) at pH of 4; fluorescence microscope and bright field images of the positive electrode (**c**) and negative electrode (**d**) at pH of 4.6; fluorescence microscope and bright field images of the positive electrode (**e**) and negative electrode (**f**) at pH of 7. At pH of 4, bacteria carried positive charges and were attracted to the negative electrode. The dyed bacteria attached to the negative electrode (**b**) were much more numerous than those that attached to the positive electrode (**a**). At pH of 4.6, the charges on the bacteria turned to neutral and similar numbers of dyed bacteria were attached to the positive (**c**) and negative (**d**) electrodes. At pH of 7, the bacteria were negatively charged, and the number of dyed bacteria attached to the positive electrode (**e**) was much more than that attached to the negative electrode (**f**).


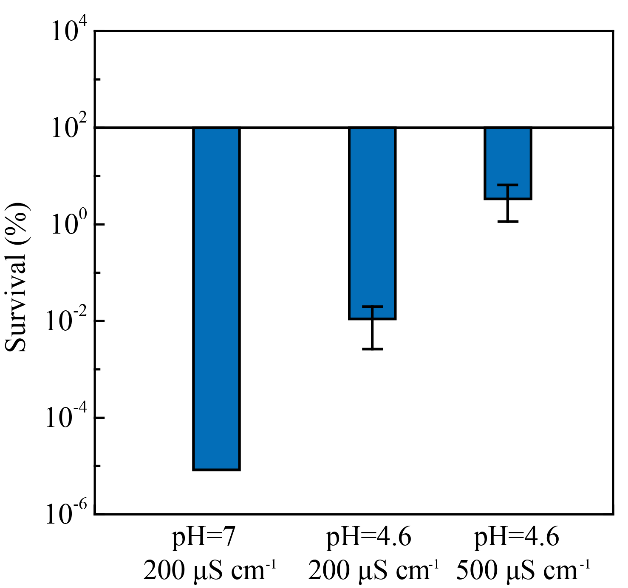


**Fig. S9. Investigation of the cell inactivation contributions of electrophoresis, dielectrophoresis and hydraulic flow.** Survival rate of *E. coli* treated by EDC at different condition. The applied voltage was fixed at 1 V and contact time was fixed at 8 s.


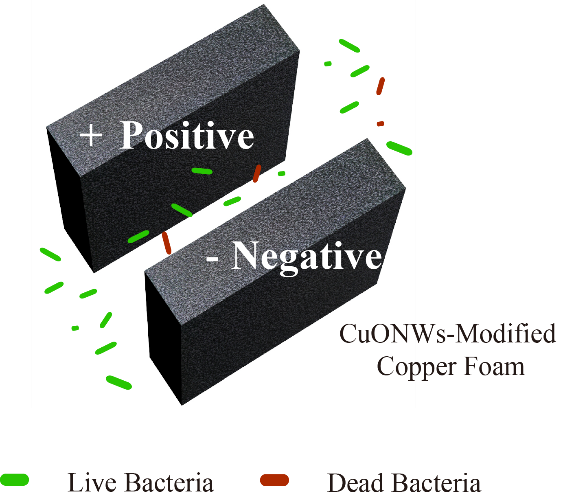


**Fig. S10.** **Cell transportation by electrophoresis and dielectrophoresis for nanowire-assisted electroporation in the batch mode.** Schematics showing the water samples treated in a batch mode EDC with designated contact time.


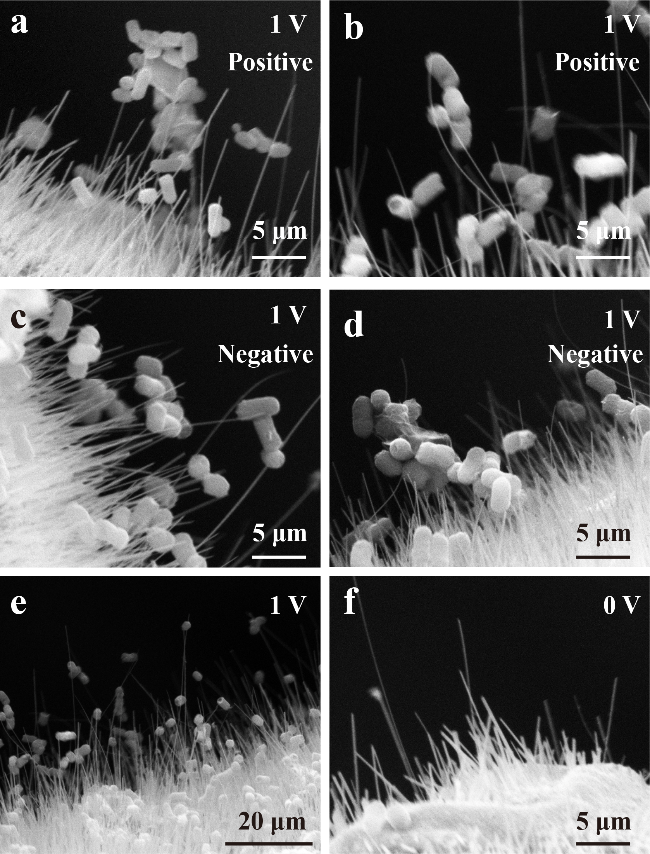


**Fig. S11.** SEM images of the positive electrode (**a, b**) and negative electrode (**c, d**) at 1 V operation with a fixed pH of 4.6. SEM image of an electrode (**e**) at 1 V operation in low magnification and SEM image of an electrode (**f**) without applied voltage. At pH of 4.6, the charges carried by the cell turned to neutral and no electrophoretic force existed. However, both positive and negative electrodes could still attract cells to the surface. This suggested that the dielectrophoretic force might also transport cells during EDC operation.


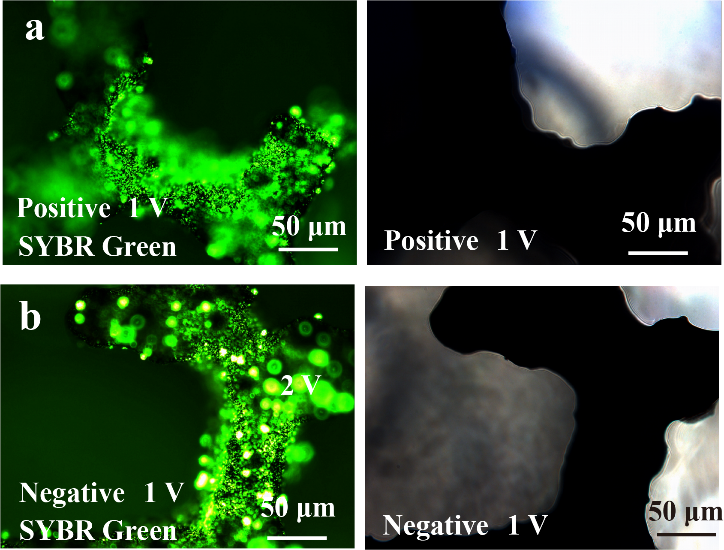


**Fig. S12. Cell transportation by dielectrophoresis for nanowire-assisted electroporation.** Fluorescence microscope and bright field images of the positive electrode (**a**) and negative electrode (**b**) at 1 V operation. At pH of 4.6, the charges carried by the cell turned to neutral and no electrophoretic force existed. However, both positive and negative electrodes could still attract cell to the surface. This suggested that the dielectrophoretic force might transport cells during EDC operation.


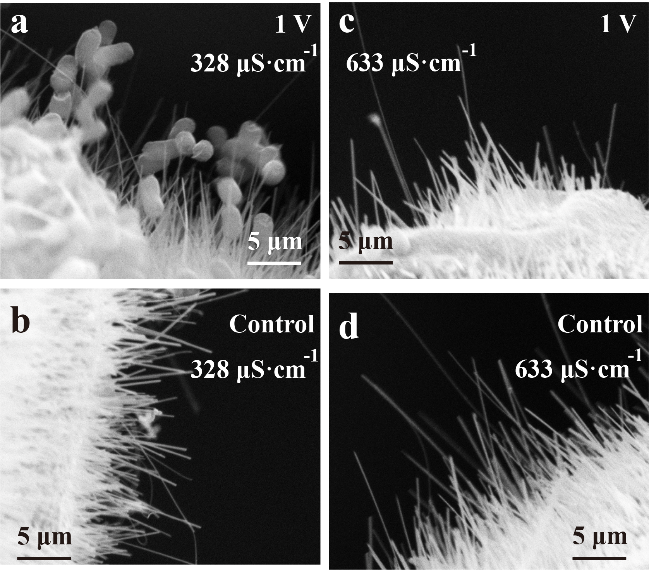


**Fig. S13. Dielectrophoretic force confirmation.** SEM images of the electrodes (**a**) with and (**b**) without applied voltage in medium with conductivity of 328 μS cm^-1^. SEM images of the electrodes (**c**) with and (**d**) without applied voltage in medium with conductivity of 633 μS cm^-1^.


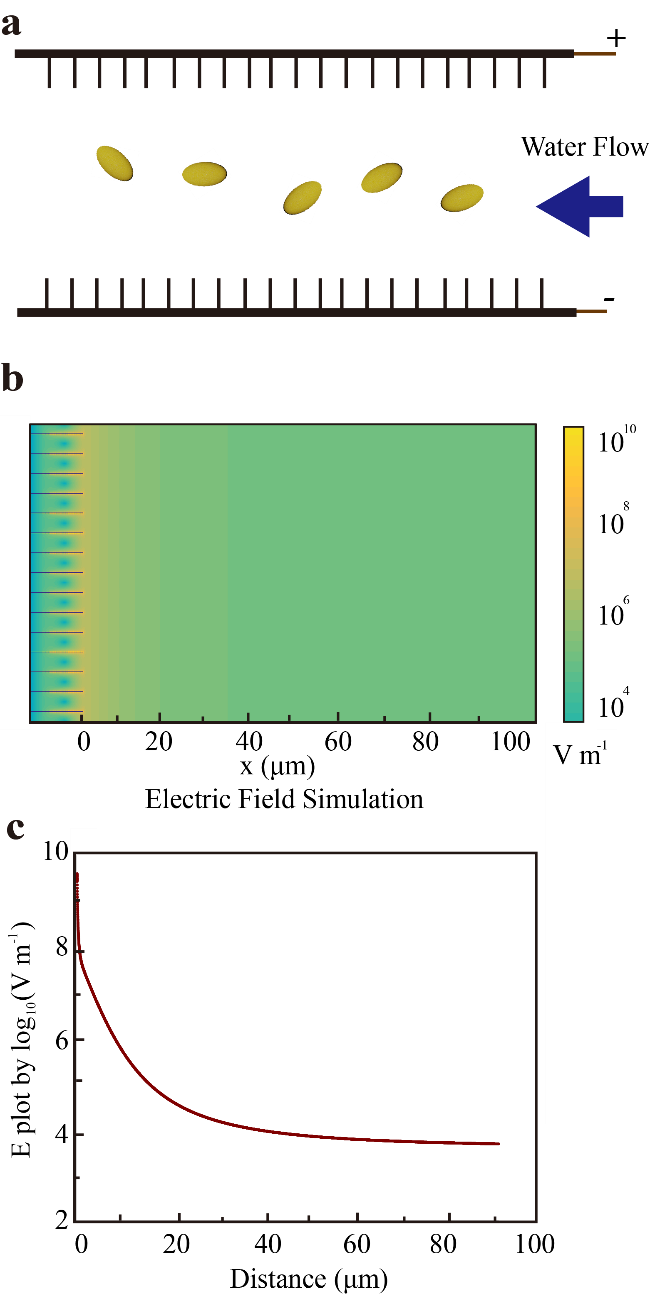


**Fig. S14. The EDC setup for computational analysis.** (**a**) Schematic of EDC equipped with two plate electrodes modified by nanowires. The distance between electrodes and the applied voltage was 100 μm and 1 V, respectively. The diameter and height of the nanowires is 10 nm and 15 μm. For each millimeter, there are 100 nanowires grown on the electrodes. (**b, c**) Simulation of electric field distribution between electrodes.

**Additional discussion 1: major forces simulation**

**1.1 Electric field strength simulation**


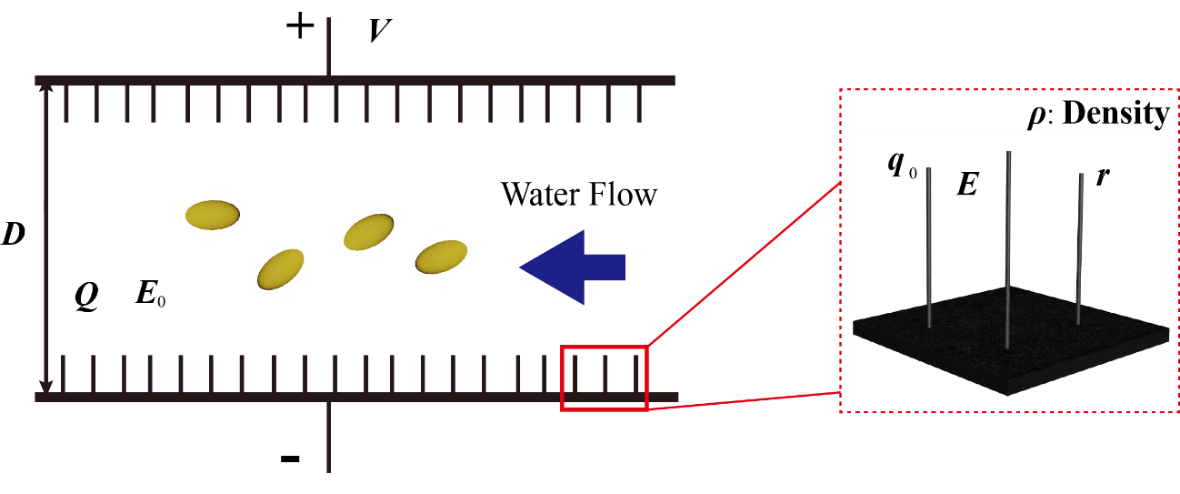


**Fig. S15.** **Schematic of simulation using a simplified EDC equipped with two plate electrodes modified by nanowires.**

To quantify the major forces, computational analysis was performed. We designed a simplified EDC equipped with two plate electrodes modified by nanowires (Fig. S15). This simplified EDC configuration was as follows: two nanowire-assisted plate electrodes were set to 100 μm apart, the voltage was 1 V, and *E. coli* contaminated water (pH of 7 and conductivity of 200 μS cm^-1^ for typical freshwater samples) flowed through the space between the two parallel electrodes as laminar flow. Under these conditions, the hydraulic force on *E. coli* cells can be ignored and the other forces (electrophoresis, dielectrophoresis, gravity, buoyancy, and Brownian force) can all be estimated. The space between two adjacent nanowires is 10 μm and the density of the nanowires is 10^4^ nanowires per mm^2^. The area of the electrode is 100 mm^2^ (10 × 10 mm).

Considering that the distance between the two electrodes (100 μm) is much greater than the nanowire scale (length, 15 μm; diameter, 10 nm) and the space between two adjacent nanowires (10 μm), the two electrodes can be considered as a parallel plate capacitor when a voltage is applied.

The electric field strength (*E_0_*) inside the parallel plate capacitor is determined by equation (1) and the total charge (*Q*) carried by electrodes can be calculated by equation (2) following Gauss' law:

$E_{0}\times D=V (1)$

$∯_{S} (E_{0}\times dS)=\frac{Q}{\varepsilon_{0}\times\varepsilon_{m}} (2)$

where *V* is the applied voltage, *S* is the area of the electrode, *ε*_0_ is the absolute dielectric constant and *ε*_m ­­_is the [relative](http://www.baidu.com/link?url=iGJu92rJ-ntTuX2CI6ZGPO29QJJqF45wVz-a6f6TFNJzttyRLrlOInp9F2Oo0v1GgfaXtqzlOrit5Ff0h3GFwx19uQUKghBCYvo6k6KYwY_) dielectric constant of the water. The total charge (*Q*) carried by the electrodes can be calculated as follows:

$E_{0}$ = 1 V / 100 μm = 10^4^ V m^-1^

Q = 10^4^ V m^-1^$\times$10^-4^ m^2^ $\times$ 8.85$\times$ 10^-12^ F m^-1^ $\times$ 80 = 7.08 $\times$10^-10^ C

In the vicinity of the electrode surface modified with conductive nanowires (density: $\rho$), the charge ($q_{0})$ carried by each nanowire is determined by equation (3):

$q_{0}\times\rho S=Q$ (3)

$q_{0}$ = 7.08 $\times$10^-10^ C / (10^-4^ m^2^$\times$ 10^4^ mm^-2^ $\times$10^6^ mm^2^ m^-2^) = 7.08 $\times$10^-16^ C


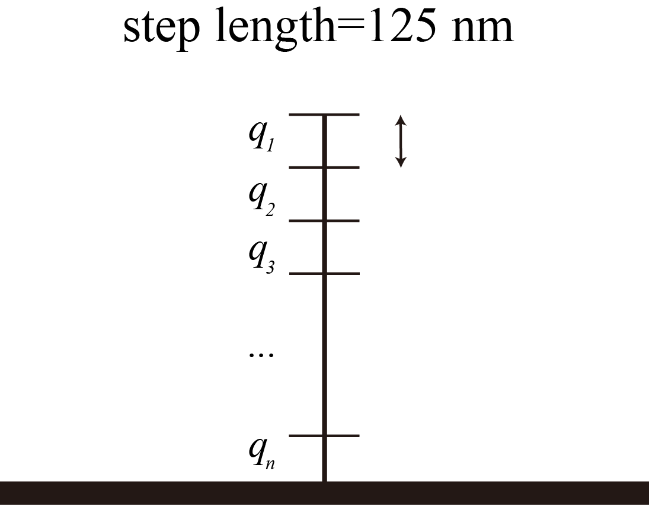


**Fig. S16.** **Schematic of single nanowire discretization in simulation.**

In the simulation, we divided one single nanowire into 40 discrete points (Fig. S16) and assumed that the charge distribution on the nanowires follows a geometric progression. The charges for each nanowire can be calculation by equations (4):

$$q_{0}=\sum_{40}^{b=1} (a^{b}\times q_{b}) (4)$$

where *a* is the common ratio of the geometric progression which equals to 0.5, $q_{b}$ is the charges carried by each discrete point and $q_{1}$=0.5$q_{0}$. We assumed that one nanowire is divided into 40 point charges.


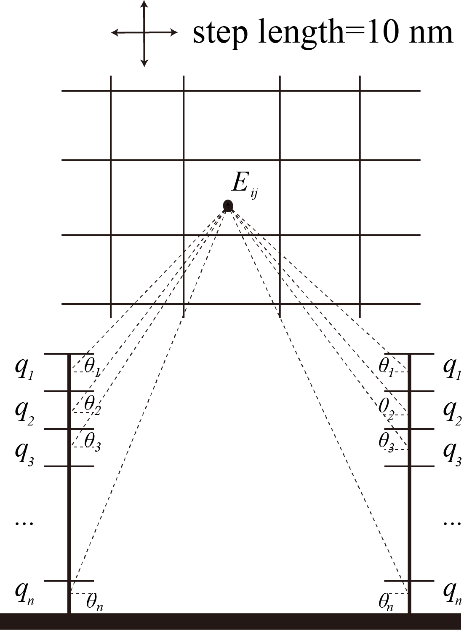


**Fig. S17.** **Schematic of electric field discretization in simulation.**

We simulated the electric field in the vicinity (100$\times$100 μm) of the tip structure of nanowire as 10^8^ discrete points (Fig. S17). We considered that the point charges of the nanowire would contribute electric field strength at each discrete point near the tip structure (100$\times$100 μm), and we simulated the electric field strength contribution of the nearest 9$\times$9 nanowires and the central nanowire as (*m, n*) (*m, n* varied from 5 to 9996) (Fig. S18).


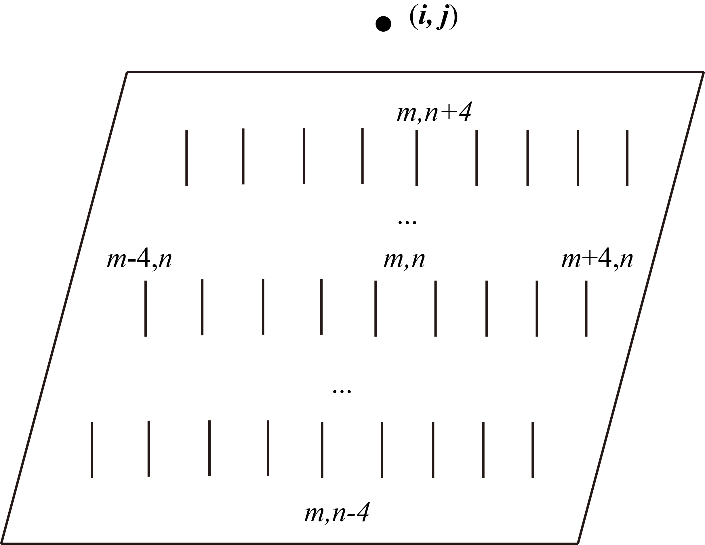


**Fig. S18.** **Schematic of electric field simulation considering the nearest 81 nanowires.**

We simulated the electric field strength of each discrete point (*i, j*) ($E_{ij}$) contributed by the nearby nanowires with central nanowire (*m, n*) by equations (5) and (6):

$$\vec{E_{i,j}^{m,n}}=\sum_{40}^{b=1} \frac{kq_{b}^{m,n}}{({r_{b}^{m,n})}^{2}} (5)$$

$$\vec{E_{ij}}=\sum_{n+4}^{n-4} \sum_{m+4}^{m-4} \vec{E_{i,j}^{m,n}} (6)$$

where $E_{i,j}^{m,n}$ is the electric field contributed by each point charge (*q_b_*) on the nanowire (*m, n*), *k* is the electrostatic force constant and $r_{b}^{m,n}$ is the distance between the discrete point (*i,j*) and discrete point (*q_b_* ) of nanowire (*m, n*). Considering that the electric field is a vector, we simulation the vertical and parallel projection and then calculation the final electric field strength.

The background strength of the electric field is *E_0_* (10^4^ V m^-1^), which is determined by the applied voltage (1 V) and the distance between the two electrodes (100 μm). Thus, the electric field strength (*E*) of a discrete point (*i, j*) near the nanowire consists of two parts: strength contributed by the nearby nanowires $E_{ij}$ and the background strength *E_0_* contributed by the opposite electrode. All the simulations were implemented by MATLAB.

**1.2 Major force simulation**

The electrophoretic force ($F_{\mathrm{EP}}$) and dielectrophoretic force ($F_{\mathrm{DEP}}$) are determined by equations (7), (8) and (9):

$F_{EP}=E_{cell}\times q_{cell}$ (7)

$F_{DEP}=2\pi\times\varepsilon_{0}\times\varepsilon_{m}\times r^{3}\times f_{CM}\times\nabla E^{2}$ (8)

$f_{CM}=\frac{\sigma_{c}-\sigma_{m}}{\sigma_{c}+2\sigma_{m}}$ (9)

where *E_cell_* is the strength of the electric field that the cell was exposed to, *ε*_0_ is the absolute dielectric constant and *ε*_m ­­_is the [relative](http://www.baidu.com/link?url=iGJu92rJ-ntTuX2CI6ZGPO29QJJqF45wVz-a6f6TFNJzttyRLrlOInp9F2Oo0v1GgfaXtqzlOrit5Ff0h3GFwx19uQUKghBCYvo6k6KYwY_) dielectric constant of the water. *f_CM_* is the Clausius-Mossotti factor, and *σ_c_* and *σ_m_* are the electrical conductivity of the microorganism (411 μS cm^-1^) and of the suspending medium (200 μS cm^-1^), respectively (1). *q_cell_* is the charge carried by one cell. *f_CM_* is calculated as follows:

*f_CM_* = _­­_(411 μS cm^-1^ – 200 μS cm^-1^)/( 411 μS cm^-1^ + 2$\times$200 μS cm^-1^) = 0.26

To calculate the charge carried by one cell, we assumed that the cell is a spherical model with radius (R) of 0.5 μm. The charge density ($c$) of the cell is 3 $\times$ 10^6^ C m^-3^ (1). The total charge (*q*) can be calculated by equation (10):

$$q_{cell}=c\times\frac{4}{3}\pi R^{3} (10)$$

*q*=3 $\times$ 10^6^ C m^-3^ $\times$ $\frac{4}{3}\pi\times$(0.5$\times$10^-6^)^3^ =1.57$\times$10^-12^ C

Gravity, buoyancy and Brownian force are estimated as 10^-14^ N (2).

**Reference**

1. Poortinga A T, Bos R, Norde W, et al. Electric double layer interactions in bacterial adhesion to surfaces. Surface Science Reports, 2002, 47(1):1-32.
2. Pethig R, Markx G H. Applications of dielectrophoresis in biotechnology. Trends in Biotechnology, 1997, 15(15):426-432.

**Table S1:  Parameter values used in the paper.**

|  | Description | Value | Unit |
| --- | --- | --- | --- |
| *D* | Distance of electrodes | 200 | μm |
| *V* | Applied voltage between electrodes | 2 | V |
| $\rho$ | Density of nanowire | 10^4^ | mm^-2^ |
| *S* | Area of electrode | 10^2^ | mm^2^ |
| *E_0_* | Electric field strength inside the parallel plate | 10^4^ | V m^-1^ |
| *Q* | Total charges carried by electrodes | 7.08$\times$10^-10^ | C |
| pH | pH of media between electrodes | 7 |  |
| *σ*_m_ | Conductivity of media between electrodes | 200 | μS cm^-1^ |
| *σ*_c_ | Conductivity of cell | 411 | μS cm^-1^ |
| *f_CM_* | Clausius-Mossotti factor determined by *σ*_m_ and *σ*_c_ | 0.26 |  |
| *L* | Length of nanowire | 15 | μm |
| *D_0_* | Diameter of nanowire | 10 | nm |
| *ε*_m_ | [Relative](http://www.baidu.com/link?url=iGJu92rJ-ntTuX2CI6ZGPO29QJJqF45wVz-a6f6TFNJzttyRLrlOInp9F2Oo0v1GgfaXtqzlOrit5Ff0h3GFwx19uQUKghBCYvo6k6KYwY_) dielectric constant of the water | 80 |  |
| *ε*_0_ | Absolute dielectric constant | 8.85$\times$10^-12^ | F m^-1^ |
| *q_0_* | Charges carried by each nanowire | 7.08$\times$10^-16^ | C |
| *q_i_* | Charges carried by each discrete point |  | C |
| *q_1_* | Charges carried by the first discrete point | 0.5 *q_0_* | C |
| *a* | Common ratio of the geometric progression | 0.5 |  |
| *E_ij_* | Electric field strength of discrete point (*i,j*) contributed by nearby nanowires |  | V m^-1^ |
| *E* | Electric field strength of discrete point (*i,j*) |  | V m^-1^ |
| *k* | Electrostatic force constant | 9$\times$10^9^ | N |
| *E_cell_* | Strength of the electric field where cell exposed in |  |  |
| *q_cell_* | Charges carried by a cell | 1.57$\times$10^-12^ | C |
| *R* | Radius of cell | 0.5 | μm |
| *c* | Charge density of cell | 3$\times$10^6^ | C m^-3^ |
| *G* | Gravity of cell | 10^-14^ | N |
| *F* | Buoyancy of cell | 10^-14^ | N |
| *B* | Brownian force | 10^-14^ | N |
